# CPP-Ts: a new intracellular calcium channel modulator and a promising tool for drug delivery in cancer cells

**DOI:** 10.1038/s41598-018-33133-3

**Published:** 2018-10-03

**Authors:** Bárbara Bruna Ribeiro de Oliveira-Mendes, Carolina Campolina Rebello Horta, Anderson Oliveira do Carmo, Gabriela Lago Biscoto, Douglas Ferreira Sales-Medina, Hortênsia Gomes Leal, Pedro Ferreira Pinto Brandão-Dias, Sued Eustáquio Mendes Miranda, Carla Jeane Aguiar, Valbert Nascimento Cardoso, André Luis Branco de Barros, Carlos Chávez-Olortégui, M. Fátima Leite, Evanguedes Kalapothakis

**Affiliations:** 1 Departamento de Biologia Geral, Instituto de Ciências Biológicas, Universidade Federal de Minas Gerais, Belo Horizonte, 31270-901 Minas Gerais Brazil; 2 Mestrado Profissional em Biotecnologia e Gestão da Inovação, Centro Universitário de Sete Lagoas, Sete Lagoas, 35701-242 Minas Gerais Brazil; 3 Faculdade de Farmácia, Universidade Federal de Minas Gerais, Belo Horizonte, 31270-901 Minas Gerais Brazil; 4 Departamento de Fisiologia e Biofísica, Instituto de Ciências Biológicas, Universidade Federal de Minas Gerais, Belo Horizonte, 31270-901 Minas Gerais Brazil; 5 Departamento de Bioquímica-Imunologia, Instituto de Ciências Biológicas, Universidade Federal de Minas Gerais, Belo Horizonte, 31270-901 Minas Gerais Brazil

## Abstract

Scorpion sting envenoming impacts millions of people worldwide, with cardiac effects being one of the main causes of death on victims. Here we describe the first Ca^2+^ channel toxin present in *Tityus serrulatus* (*Ts*) venom, a cell penetrating peptide (CPP) named CPP-Ts. We show that CPP-Ts increases intracellular Ca^2+^ release through the activation of nuclear InsP3R of cardiomyocytes, thereby causing an increase in the contraction frequency of these cells. Besides proposing a novel subfamily of Ca^2+^ active toxins, we investigated its potential use as a drug delivery system targeting cancer cell nucleus using CPP-Ts’s nuclear-targeting property. To this end, we prepared a synthetic CPP-Ts sub peptide^14–39^ lacking pharmacological activity which was directed to the nucleus of specific cancer cell lines. This research identifies a novel subfamily of Ca^2+^ active toxins and provides new insights into biotechnological applications of animal venoms.

## Introduction

Scorpion sting envenoming has been officially established as a neglected public health issue by the World Health Organization^1^. Over 1.5 million of scorpion stings and more than 2,600 deaths occur annually worldwide^2^. Besides intense local pain, severe envenoming can lead to multi-organ failure, including the potentially lethal cardiogenic shock and pulmonary oedema^3^.

The deleterious effects of scorpion venoms are related to the synergistic action of a variety of components, especially the highly toxic neurotoxins that modulate Na^+^, K^+^, Ca^2+^ and Cl^−^ currents, thereby causing depolarization of excitable cells and massive release of neurotransmitters^4,5^. Toxins that act on plasma membrane K^+^ channels (KTx), although abundant, have little toxicological significance for mammals^6^. Indeed, most of the symptoms of scorpionic envenomation have been associated with the binding of toxins to specific sites of plasma membrane Na^+^ channels (NaTx)^7^.

The most venomous scorpion in South America, *Tityus serrulatus* (*Ts*) (Lutz and Mello Campos, 1922), presents KTx and NaTx, the toxins that have the greatest medical importance in envenomation^8^. However, toxins specific to Ca^2+^ (CaTx) and Cl^-^ (ClTx) channels remain undetected in this species, as with most scorpions^9^. Therefore, research identifying whether *Ts* venom mixture contains these toxins and if so, their mechanism of action, is important to understand *Ts* envenomation process and a necessary step in the search for therapeutic solutions.

The CaTxs act on voltage-dependent plasma membrane or intracellular Ca^2+^ channels^10–12^. The latter pertains to the scorpionic calcine family, which is interesting because these toxins belong to the cell penetrating peptides (CPPs) class^13^. CPPs have been subject of much attention in studies aiming to develop non-invasive drug delivery systems. Indeed, the cellular membrane is very effective in its role as a selectively permeable barrier, and many drugs that fail to cross the cell membrane barrier have been successfully delivered to the site of action when fused to CPPs^14,15^.

This work describes for the first time a scorpionic toxin that modulates intracellular Ca^2+^ channels, in the *Ts* venom, named CPP-Ts. This toxin is involved in the cardiac symptomatology of *Ts* envenomation by directly affecting cardiomyocytes through a novel mechanism of action for animal toxins. Additionally, CPP-Ts presents nuclear internalization properties in specific cancer cell lines and is unable to cross the cell membrane of normal cell lines, which highlights this peptide as a promising and specific tool for intranuclear delivery to cancer cells.

## Results

### CPP-Ts is a new Ts toxin and is distinct from other scorpion calcines

**Figure 1:**
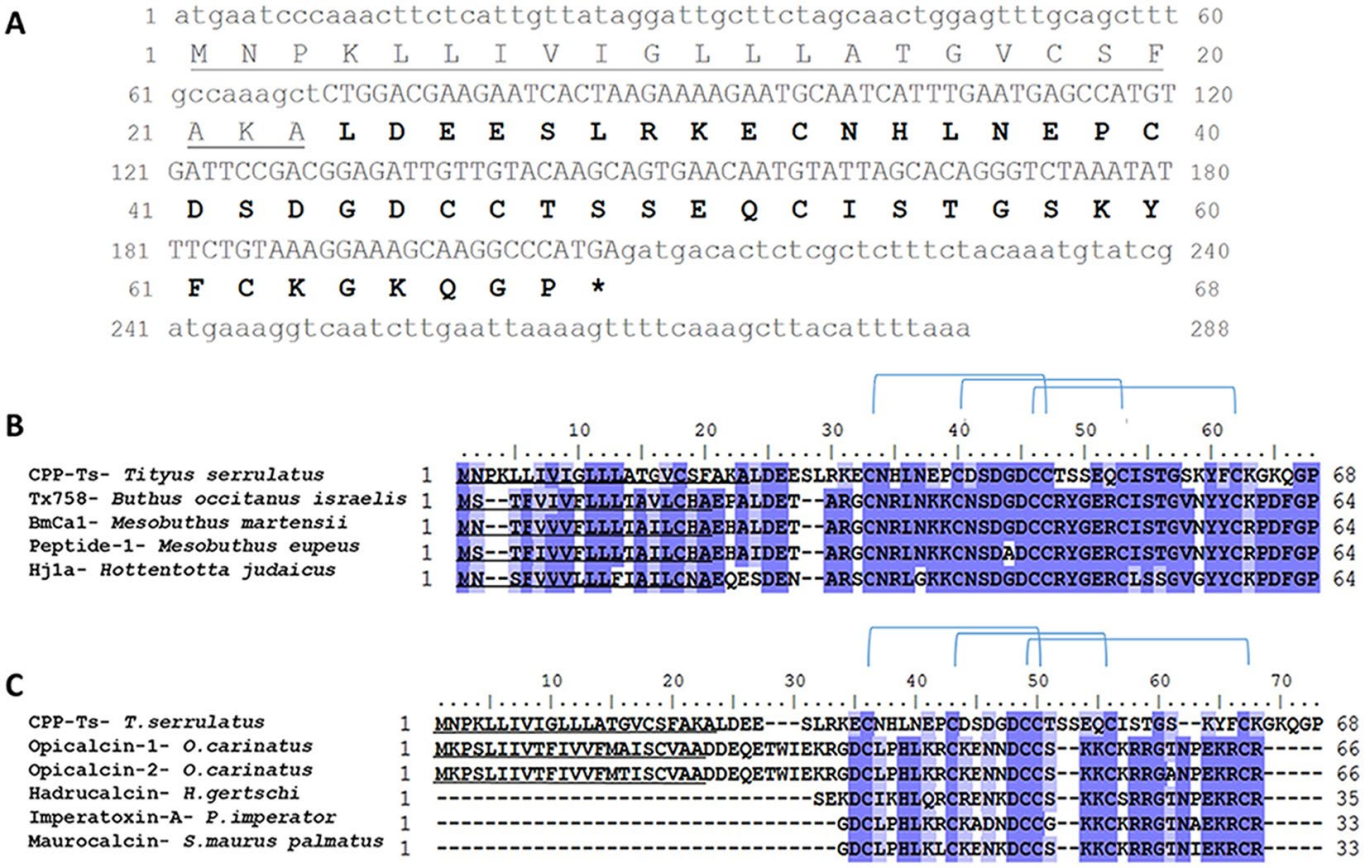
Sequence of CPP-Ts from *T*. *serrulatus* venom and alignment with other scorpionic Ca^2+^ channel toxins. (**A**) Figure shows cDNA and predicted amino acid sequences of CPP-Ts (GenBank MH061344). The signal peptide sequence is underlined. Mature protein sequence is represented by the bolded amino acids and the capital nucleotides. Stop codon is represented by an asterisk. (**B**–**C**) Conserved residues are marked in dark blue, similar ones in light blue, and cysteine forming disulfide bonds are connected by lines. The signal peptide sequence is underlined. (**B**) Alignment of *T*. *serrulatus* CPP-Ts amino acid sequence with the highest similarity sequences available in the database (60% similarity). Those are Tx758 from *Buthus occitanus israelis* (UniProt B8XH22), BmCa1 from *Mesobuthus martensii* (UniProt Q8I6X9), Peptide-1 from *Mesobuthus eupeus* (UniProt P86399), and Hj1a from *Hottentotta judaicus* (GenBank ADY39527.1). (**C**) CPP-Ts amino acid sequence alignment with toxins from the scorpionic calcine group (<40% similarity): Opicalcin-1 (UniProt P60252) and Opicalcin-2 (UniProt P60253) from *Opistophthalmus carinatus*, Hadrucalcin from *Hadrurus gertschi* (GenBank ACC99422.1), Imperatoxin-A from *Pandinus imperator* (UniProt P59868), and Maurocalcin from *Scorpio maurus palmatus* (UniProt P60254).

CPP-Ts was found for the first time as a component of *Ts* venom through transcriptome analysis. Both nucleotide and deduced amino acid sequences are shown in Fig. 1A. The complete toxin is composed of 288 bp, and the predicted amino acid sequence is amino acid 68 residues in length (CPP-Ts amino acid sequence: MNPKLLIVIGLLLATGVCSFAKALDEESLRKECNHLNEPCDSDGDCCTSSEQCISTGSKYFCKGKQGP). The signal peptide comprises the 23 first amino acid residues of the protein. Thus, mature CPP-Ts has 45 amino acids (Fig. 1A). Mature CPP-Ts has a pI of 4.6 and Mw of 4943.38 Da, is hydrophilic and carries a global acid charge, which distinguishes it from other toxins from *Ts* venom that are mainly basic^16^. CPP cDNA and protein sequences are available in GenBank database under the accession number MH061344.

In the BLASTp search against the UniProtKB/Swiss-Prot database, CPP-Ts showed approximately 60% similarity to toxins from other scorpions that were classified as active on Ca^2+^ channels. These similarities were also found in a nucleotide analysis. The most similar sequences were the toxins Tx758 from *Buthus occitanus israelis* (UniProt B8XH22), BmCa1 from *Mesobuthus martensii* (UniProt Q8I6X9), Peptide-1 from *Mesobuthus eupeus* (UniProt P86399), and Hj1a from *Hottentotta judaicus* (GenBank ADY39527.1). However, none of these toxins have been previously biochemically or pharmacologically characterized. Despite the amino acid sequence conservation, the CPP-Ts polypeptide differs the most from the other putative CaTx (Fig. 1B). Interestingly, all the cysteines (C), which form the disulfide bonds in the proteins, are conserved among all sequences. CPP-Ts’s disulfide bond formation occurs between the residues C10-C24, C17-C30, and C23-C39 in the mature protein, following similar toxins with known structures (UniProt Q8I6X9; UniProt B8XH22; UniProt P86399). The mature CPP-Ts sequence of 45 amino acids with the referred disulfide bonds was chemically synthesized and used in biological assays.

We also evaluated the similarity of CPP-Ts to other calcine scorpion toxins known to affect Ryanodine Receptor (RyR) Ca^2+^ channels (Fig. 1C)^13,17–19^. The aligned sequences were: Opicalcin-1 (UniProt P60252) and Opicalcin-2 (UniProt P60253) from *Opistophthalmus carinatus*; Hadrucalcin from *Hadrurus gertschi* (GenBank ACC99422.1); Imperatoxin-A from *Pandinus imperator* (UniProt P59868); and Maurocalcin from *Scorpio maurus palmatus* (UniProt P60254). Although amino acids such as the cysteines are conserved, the average similarity among the sequences is lower than 40% (marked in blue on Fig. 1C).

### CPP-Ts increases Ca^2+^ transient in neonatal rat cardiomyocytes

**Figure 2:**
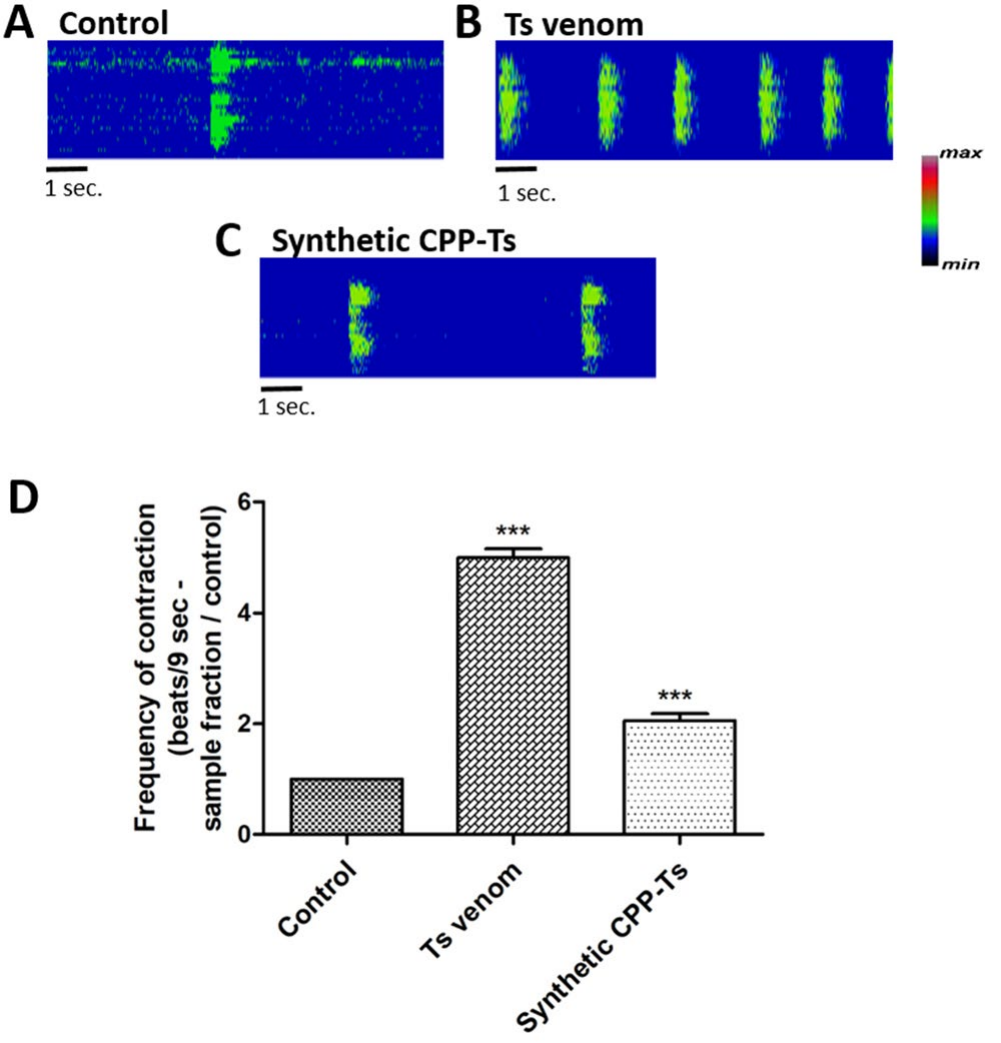
Synthetic CPP-Ts and *T*. *serrulatus* venom alter the Ca^2+^ transient in cardiomyocytes. Neonatal rat cardiomyocytes were used for the functional analysis of *Ts* venom and synthetic CPP-Ts. Ca^2+^ was monitored with Fluo-4/AM using confocal line-scanning microscopy (magnification x63). (**A**–**C**) Global Ca^2+^ transient in cardiomyocytes. Cells were examined immediately after treatment with B: *Ts* venom (12.8 µg/ml), C: synthetic CPP-Ts (2 µg/ml). Images are pseudocolored according to the color scale. (**D**) Global Ca^2+^ transient analysis measured by the number of contractions over 9 seconds. Treatments with *Ts* venom and synthetic CPP-Ts significantly increased the contraction frequency in neonatal rat cardiomyocytes, when compared to control (F_3,19_ = 133.4, p = 6.08 e-13; t_TsV_ = 25.12, df_TsV_ = 19, p_TsV_ = 4.44 e-16; t_synth. CPP_ = 8.407, df_synth. CPP_ = 19, p_synth. CPP_ = 7.95 e-08). All values correspond to the mean ± S.E.M (n = 20 cells per treatment) of three independent experiments. Statistical analyses were performed using Repeated Measures ANOVA followed by paired t-tests corrected with Bonferroni procedure.

Synthetic CPP-Ts and *Ts* venom altered the amplitude and kinetics of Ca^2+^ transients in cardiomyocytes. They provoked a significant increase in Ca^2+^ release frequency, thus increasing the cells contraction frequency. Synthetic CPP-Ts doubled cardiomyocytes’ contraction frequencies, whereas *Ts* venom promoted a 5 times increase (Fig. 2A–D).

### CPP-Ts has nuclear localization

**Figure 3:**
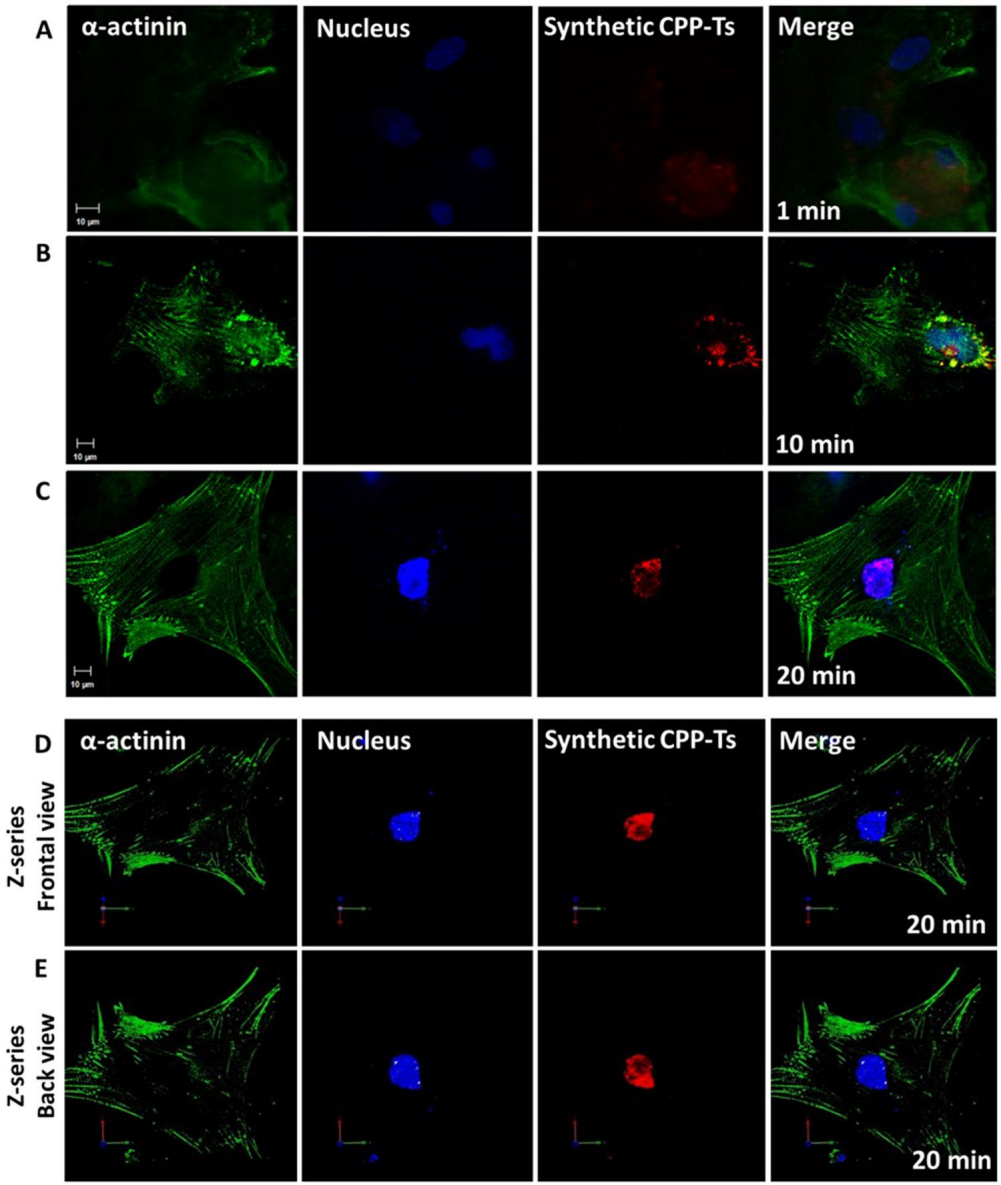
Intranuclear localization of CPP-Ts in cardiomyocytes over time. Neonatal rat cardiomyocytes were double labeled with α-actinin (green) and the nucleus marker TO-PRO-3 (blue). CPP-Ts was labeled with Alexa 555 nm (red). (**A**–**C**) Confocal immunofluorescence, in single plan, shows: (**A**) Diffuse intracellular localization of CPP-Ts after 1 min of treatment; (**B**) CPP-Ts concentrated in the perinuclear region after 10 min of treatment; (**C**) Intranuclear localization of CPP-Ts after 20 min of treatment. It is noteworthy that CPP-Ts is driven to the nuclear region over time. (**D**,**E**) Three-dimensional images from confocal microscopy analyzed by Z-series on Volocyte program shows colocalization of CPP-Ts and the nucleus. (**D**) Cell frontal view with nucleus labeling adjusted for solid surface. (**E**) Cell back view with nuclear labeling adjusted for solid surface. It is noticeable that TO-PRO3 fluorescence completely covers Alexa 555, in all three dimensions (**D**,**E**), thus validating the intranuclear localization of CPP-Ts.

We evaluated the subcellular localization of synthetic CPP-Ts in multiple internalization times. It showed a diffuse localization in cardiomyocytes with an internalization time of 1 min (Fig. 3A), at 10 min it was concentrated in the perinuclear region (Fig. 3B), and it was finally almost completely internalized into the cell nucleus within 20 min (Fig. 3C). The cellular images collected in Z-series and analyzed by the Volocyte program showing CPP-Ts colocalized with nuclear markers (Fig. 3D–E), thus validating the intranuclear localization of CPP-Ts. Therefore, CPP-Ts crosses the cellular membrane and is quickly directed to the intranuclear region of cardiomyocytes (Fig. 3).

### Nuclear InsP3 receptors are involved in Ca^2+^ modulation induced by CPP-Ts and *Ts* venom

**Figure 4:**
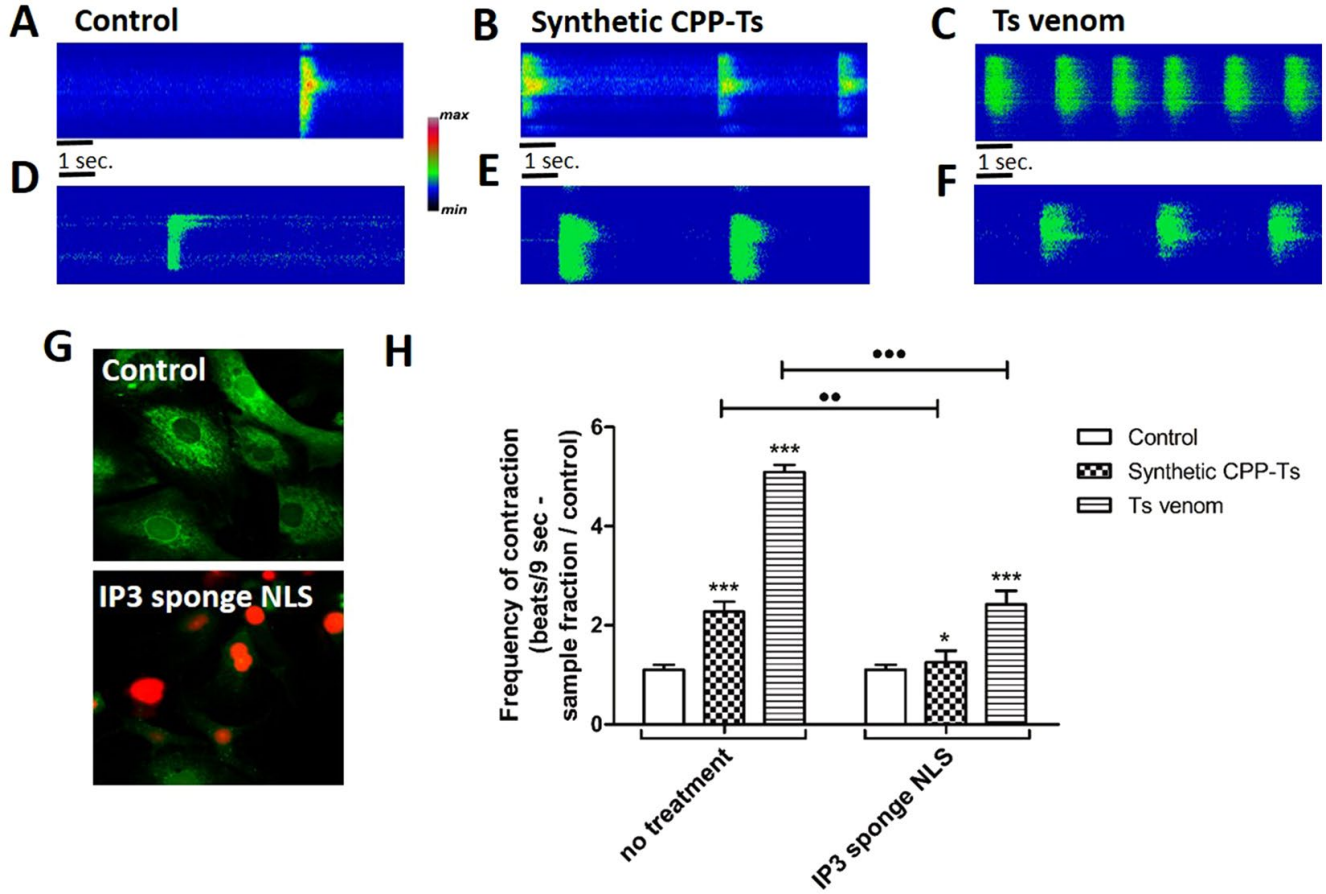
Effects of synthetic CPP-Ts and *Ts* venom in cardiomyocytes are reduced in cells transfected with nuclear InsP3 sponge NLS virus. Neonatal rat cardiomyocytes were transfected with IP3 sponge NLS virus and then treated with synthetic CPP-Ts or *Ts* venom. Experiments were conducted by monitoring of Ca^2+^ with Fluo-4/AM using confocal line-scanning microscopy. Global Ca^2+^ transient was examined immediately after treatment with CPP-Ts (2 µg/ml) or *Ts* venom (12.8 µg/ml) in untransfected cardiomyocytes (**A**–**C**) or in cardiomyocytes transfected with nuclear IP3 sponge NLS virus (**D**–**F**). Images are pseudocolored according to the color scale. (**G**) Representative image of both untransfected (control) and transfected cells. Intracellular Ca^2+^ is marked in green by Fluo-4/AM and the virus nuclear transfection is labeled in red. (**H**) Global Ca^2+^ transient analysis measured by the number of contractions over 9 seconds. Untransfected cells, treated with CPP-Ts and *Ts* venom (F_2,19_ = 616.9, p = 0; t_synth. CPP_ = 9.097, df_synth. CPP_ = 19, p_synth. CPP_ = 2.36 e-08***; t_TsV_ = 40.04, df_TsV_ = 19_,_ p_TsV_ = 0***) significantly increased the contraction frequency in neonatal rat cardiomyocytes, compared to control. However, in cardiomyocytes transfected with IP3 sponge NLS virus (F_2,19_ = 30.82, p = 1.08 e-06; t_synth. CPP_ = 2.378, df_synth. CPP_ = 19, p_synth. CPP_ = 0.0281*; t_TsV_ = 8.208, df_TsV_ = 19_,_ p_TsV_ = 1.14 e-07***), there is a significant decrease in contraction frequency compared to the untransfected group after treatment with both CPP-Ts (t = 3.454, df = 38, p = 0.0014^••^) or *Ts* venom (t = 3.42, df = 38, p = 4.44 e-16^•••^). This indicates that nuclear Ca^2+^ has an important role in the increase of global Ca^2+^ transient provoked by CPP-Ts and *Ts* venom in cardiomyocytes. All values are the mean ± S.E.M. (n = 20 cells per treatment) of three independent experiments. Statistical analyses were performed using Repeated Measures ANOVA followed by paired or unpaired t-tests corrected with Bonferroni procedure.

Nuclear inositol 1,4,5-trisphosphate (InsP3) plays a crucial role in cardiomyocyte functions^20^. We used cardiomyocytes transfected with InsP3 sponge NLS virus to verify CPP-Ts and *Ts* venom action (Fig. 4). Increased Ca^2+^ transient was observed in non-transfected cardiomyocytes incubated with synthetic CPP-Ts (2 µg; ***p = 2.36 e-08) and *Ts* venom (12.8 µg; ****p = 0) (Fig. 4A–C,H). Interestingly, transfected cardiomyocytes showed a significant decrease in contraction frequency compared to the untransfected cells after treatment with synthetic CPP-Ts (^••^ p = 0.0014) or *Ts* venom (^•••^ p = 4.44 e-16) (Fig. 4D–F,H). This result suggests that nuclear Ca^2+^, through InsP3 receptor (InsP3R) activation, plays an important role in Ca^2+^ transient promoted by CPP-Ts and *Ts* venom.

### ^99m^Tc-CPP-Ts has uptake affinity for specific organs

Thin layer chromatography (TLC) was used to determine the radiochemical efficiency of the labeling with Technetium-99m to prepare ^99m^Tc-CPP-Ts. The results indicated high radiochemical yield (95.2 ± 2.4%). The complex ^99m^Tc-CPP-Ts showed high stability in saline and plasma even after long periods (over 90% −24 h; Supplementary Fig. 1), indicating that it had favorable characteristics for further biodistribution assays.

**Figure 5:**
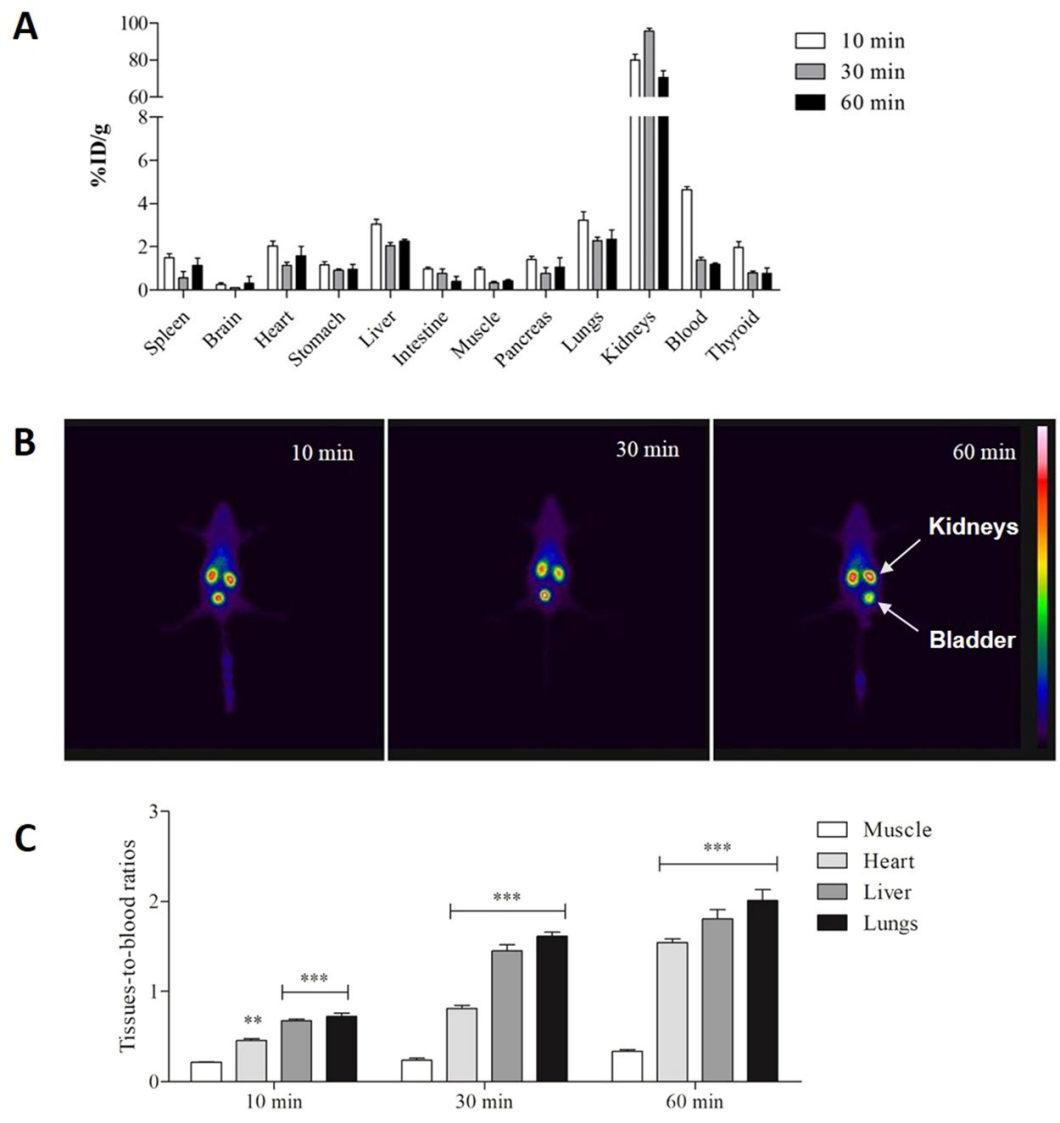
Biodistribution of synthetic ^99m^Tc-CPP-Ts. (**A**) ^99m^Tc-CPP-Ts (3.7 MBq) was intravenously injected in Swiss mice (n = 7, 6–8 weeks old, 24–28 g). After 10, 30, and 60 min post-injection, the radioactivity was measured in liver, spleen, kidneys, stomach, heart, lungs, blood, muscle, thyroid, intestine, brain, and pancreas. The results are expressed as the percentage of injected dose/g of tissue (%ID/g). (**B**) Representative scintigraphic images of mice (n = 3, 6–8 weeks old, 24–28 g) injected with 11 MBq ^99m^Tc-CPP-Ts after 10, 30, and 60 min, showing a very high kidney uptake. (**C**) Comparing the tissues-to-blood ratios, there is an increasing ratio over time for heart, lungs, and liver when compared to muscle, a non-specific tissue. After 60 min, ratios reached values higher than 1.5, indicating that such organs had more than 50% of the ^99m^Tc-CPP-Ts uptake compared to blood (_organ_F_3,72_ = 282.6, p = 0; _time_F_2,72_ = 277, p = 0; t_Heart10′_ = 11.90, df_Heart10′_ = 6, p_Heart10′_ = 2.13 e-05**; t_Liver10′_ = 34.91, df_Liver10′_ = 6, p_Liver10′_ = 3.6 e-08***; t_Lungs10′_ = 15.59, df_Lungs10′_ = 6, p_Lungs10′_ = 4.40 e-06***; t_Heart30′_ = 16.44, df_Heart30′_ = 6, p_Heart30′_ = 3.22 e-06***; t_Liver30′_ = 20.73, df_Liver30′_ = 6, p_Liver30′_ = 8.20 e-07***; t_Lungs30′_ = 38.13, df_Lungs30′_ = 6, p_Lungs30′_ = 2.17 e-08***; t_Heart60′_ = 29.47, df_Heart60′_ = 6, p_Heart60′_ = 1.01 e-7***; t_Liver60′_ = 15.93, df_Liver60′_ = 6, p_Liver60′_ = 3.88 e-06***; t_Lungs60′_ = 15.47, df_Lungs60′_ = 6, p_Lungs60′_ = 4.61 e-6***). Data are expressed as mean S.E.M of two independent experiments. Statistical analyses were performed using Two-way analysis of variance followed by unpaired t-tests corrected with Bonferroni procedure.

Biodistribution of ^99m^Tc-CPP-Ts after intravenous administration in mice is shown in Fig. 5. High uptake was observed in the kidneys as this is probably the main elimination route, and this is consistent with the hydrophilic nature of ^99m^Tc-CPP-Ts. Because the thyroid only accumulates free ^99m^Tc, the low uptake of ^99m^Tc-CPP-Ts suggests that the complex is highly stable, as predicted by the *in vitro* stability assay detailed above.

Scintigraphic images of these mice corroborated the biodistribution findings, showing a very high uptake by the kidneys (Fig. 5B). Additionally, some tissues, such as heart, liver, and lungs showed significantly higher uptake than other organs, indicating the preference of ^99m^Tc-CPP-Ts to those tissues. Tissues-to-blood ratios increased over time for heart, lungs, and liver (Fig. 5C). After 60 min, ratios reached values higher than 1.5, indicating that these organs had more than 50% of the ^99m^Tc-CPP-Ts uptake compared to blood. Thus, ^99m^Tc-CPP-Ts has high affinity to the heart, liver, and lungs, since the uptake of the complex by these organs is not a consequence of blood circulation. In contrast, the uptake values of a non-specific tissue such as muscle showed that the ratios of all timeframes were much lower than those presented by the heart, lungs, and liver (Fig. 5C).

### CPP-Ts is not lethal to mice, and anti-CPP-Ts serum neutralizes toxic effects of the *Ts* venom

Anti-CPP-Ts serum reactivity was tested against synthetic CPP-Ts and *Ts* venom using rabbit pre-immune serum as the control. While anti-CPP-Ts serum recognized the CPP-Ts toxin, only small interaction with *Ts* venom was detected by ELISA (Supplementary Fig. 2). The low reactivity may be related to the low concentration of CPP-Ts in *Ts* venom.

Anti-CPP-Ts serum neutralized 50% of 2 LD_50_ and 75% of 1.5 LD_50_ of *Ts* venom in mice (Supplementary Table 1). This indicates that, in spite of its low concentration, CPP-Ts plays an important role in the lethality of Ts envenomation. However, *in vivo* toxicity assays showed that synthetic CPP-Ts toxin was not lethal to mice (100% survival), even when a high dose was used (72.5 µg/20 g that is equivalent to 5.5 LD_50_ of *Ts* venom). Considering that anti-CPP-Ts serum reduced the mortality of animals exposed to the whole venom, this toxin probably acts synergistically with other venom components.

### Sub peptide^14–39^ has nuclear localization but lacks pharmacological activity

**Figure 6:**
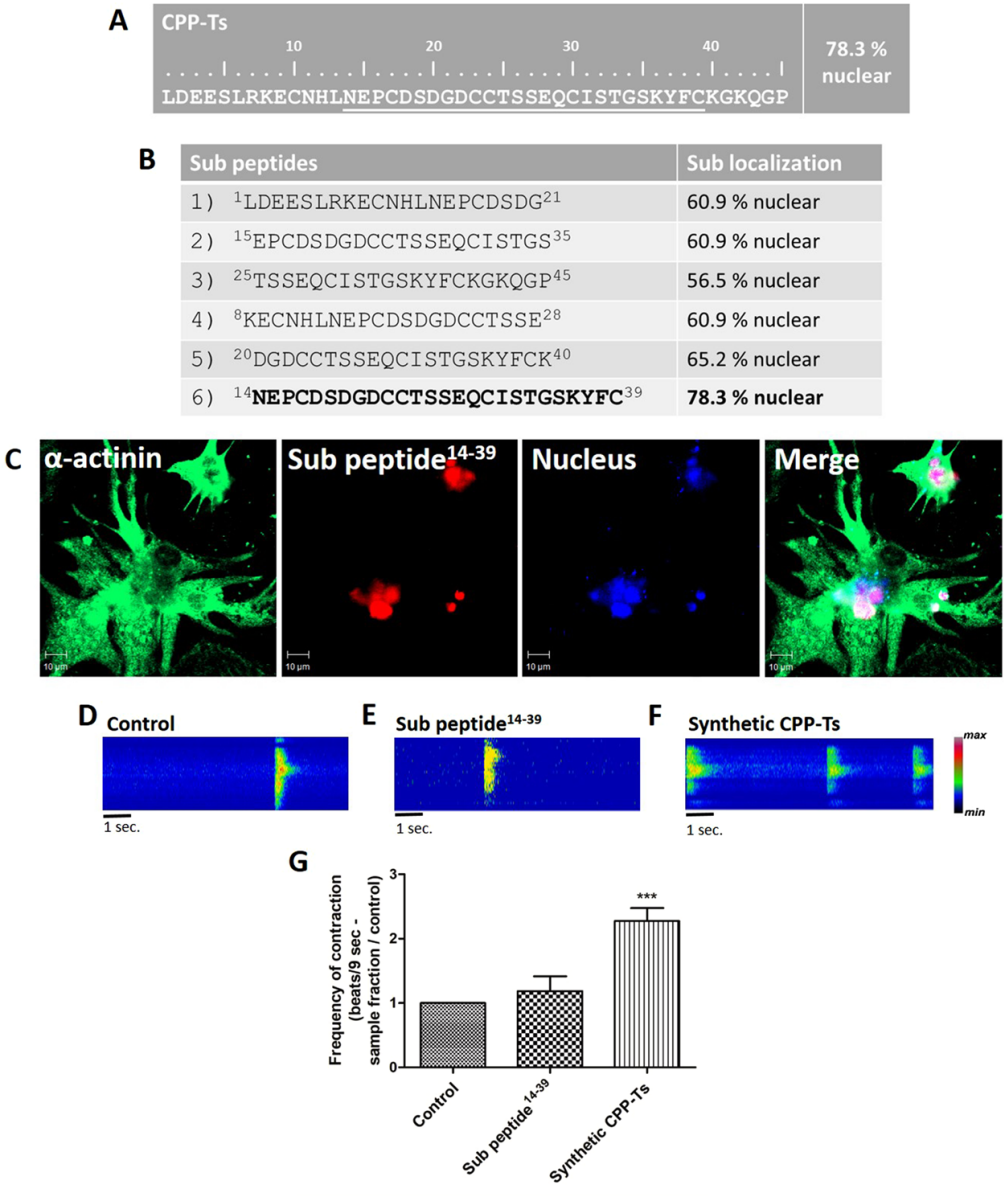
*In silico* prediction of the sub cellular localization and activity of sub peptide^14–39^ that carries the nuclear internalization property but lacks CPP-Ts biological activity. Mature CPP-Ts and six sub peptide sequences were subjected to the sub cellular localization prediction analysis using the PSORT II software. (**A**) CPP-Ts was accurately predicted (78.3%) to have a nuclear localization. (**B**) Predictions of sub cellular localization of the sub peptides varied from 56.5% to 78.3%. The sub peptide^14–39^ was the only one that carried the full CPP-Ts nuclear internalization properties. (**C**) Single plane images of neonatal rat cardiomyocytes from confocal microscopy. Merged image of cytoskeleton (green, α-actinin, 488 nm), sub peptide^14–39^ (red, Alexa 555, 555 nm) and nucleus (blue, TO-PRO3, 633 nm) labeling showing intranuclear localization of sub peptide^14–39^ after 20 min of treatment. (**D**–**G**) Global Ca^2+^ transient analysis of cardiomyocytes stained with Fluo-4/AM using confocal line-scanning microscopy. Images are pseudocolored according to the color scale. Cells were examined immediately after treatment with sub peptide^14–39^ (2 µg/ml) or synthetic CPP-Ts (2 µg/ml) used as positive control. The frequency of cell contractions was altered by the treatment with synthetic CPP-Ts, but it was not significantly altered by the treatment with the sub peptide^14–39^ (F_2,29_ = 44.76, p = 1.36 e-09; t_sub pep_ = 1.705, df_sub pep_ = 29_,_ p_sub pep_ = 0.0989; t_synth. CPP_ = 8.572, df_synth. CPP_ = 29, p _synth. CPP_ = 1.92 e-09). All values correspond to the mean ± S.E.M (n = 20 cells per treatment) of three independent experiments. Statistical analyses were performed using Repeated Measures ANOVA followed by paired t-tests corrected with Bonferroni procedure.

The cellular sub localization of CPP-Ts was predicted using the PSORT-II program with a 78.3% nuclear localization probability (Fig. 6A). To identify sub peptides that carry the necessary information for nuclear internalization of the molecule, we designed sub peptides from the mature CPP-Ts sequence for *in silico* analyses (Fig. 6B). Five were less likely to undergo nuclear internalization when compared to the complete CPP-Ts (Fig. 6B). However, the sub peptide 6, corresponding to CPP-Ts amino acids 14 to 39 (sub peptide^14–39^), presented the same nuclear localization probability (78.3%) as the complete toxin (Fig. 6A,B). This sub peptide was then synthesized.

Cardiomyocyte internalization assays showed that the sub peptide^14–39^ is directed to the nuclear region within 20 min, similarly to synthetic CPP-Ts (Fig. 6C). However, unlike the complete toxin, it did not alter the Ca^2+^ transient and the contraction frequency (Fig. 6D–G). Thus, the sub peptide^14–39^ carries the nuclear internalization properties of CPP-Ts, but not its pharmacological activity.

In addition, sub peptide^14–39^ does not have cytotoxic effects in cardiomyocytes, as well as synthetic CPP-Ts and *Ts* venom (Supplementary Fig. 3).

### CPP-Ts has selective internalization properties in primary culture and cancerous cells

**Figure 7:**
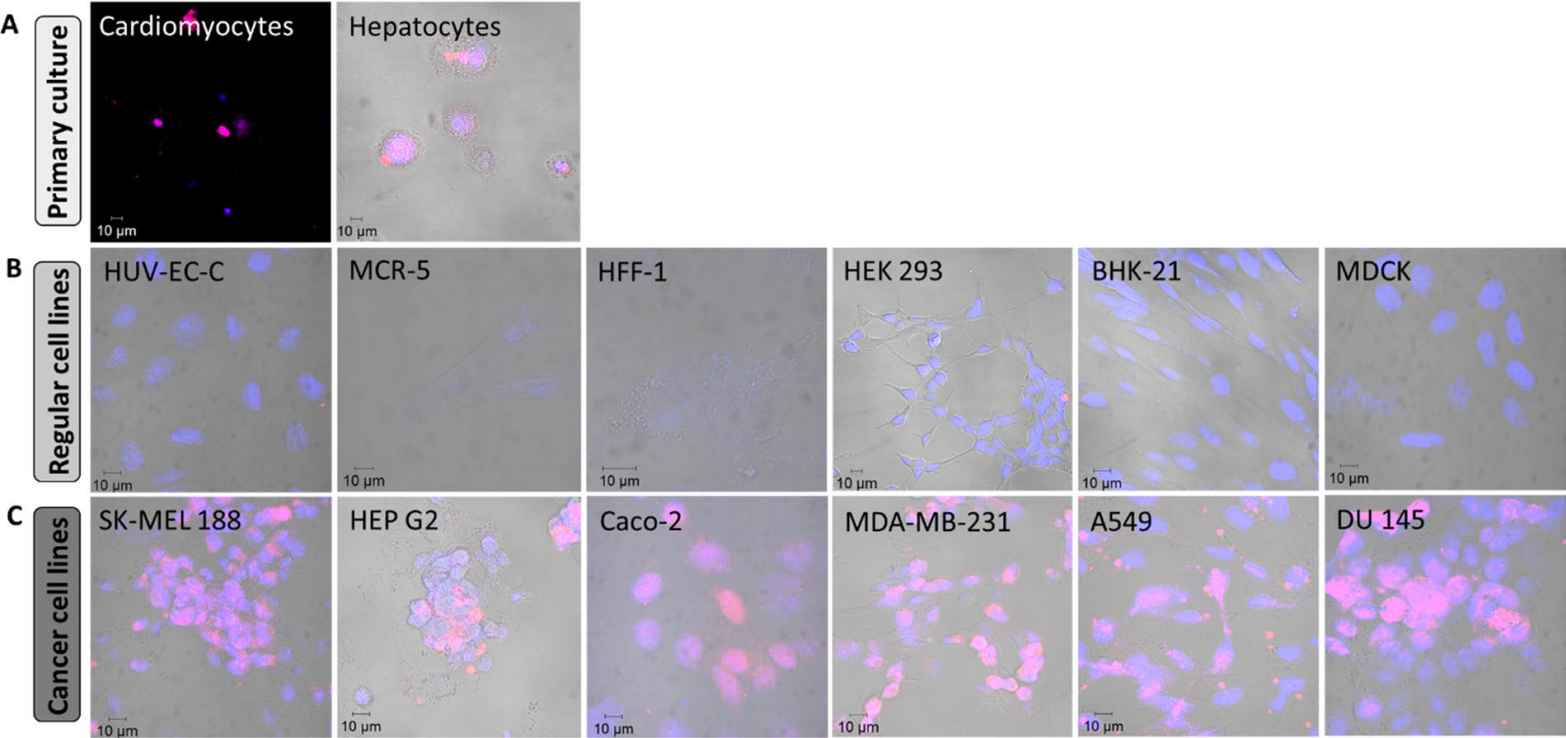
Sub peptide^14–39^ selectivity cell internalization. The internalization property of sub peptide^14–39^ (20 µg/ml) was investigated in different cell lines and primary cultures. Confocal microscopy images represent the nucleus in blue (TO-PRO3, 633 nm) and sub peptide^14–39^ in red (Alexa 555, 555 nm). Sub peptide^14–39^ is able to entry in primary culture cells as cardiomyocytes and hepatocytes (**A**), however it is not able to entry in six normal immortalized cell lines tested: HUV-EC-C, MCR-5, HFF-1, HEK-293, BHK- 21 and MDCK (**B**). Interestingly, sub peptide^14–39^ internalizes in six cancer cell lines tested: SK-MEL-188, HEP G2, Caco-2, MDA-MB-231, A549 and DU 145 (**C**). The images represent two coverslips (n = 300 cells) for each cell line tested.

We analyzed the cellular internalization properties of sub peptide^14–39^ in multiple cell lines (Fig. 7). The sub peptide^14–39^ presented nuclear localization in all primary cultures tested (rat cardiomyocytes and rat hepatocytes) (Fig. 7A). However, the sub peptide was unable to cross the cell membrane in all the six normal immortalized cell lines (HUV-EC-C, HFF-1, MCR-5, HEK-293, BHK-21 and MDCK) (Fig. 7B). Surprisingly, sub peptide^14–39^ was internalized and directed to the intranuclear region in all six neoplastic cell lines analyzed (SK-MEL-188, HEP G2, Caco-2, MDA-MB-231, A549 and DU 145) (Fig. 7C). Therefore, the sub peptide^14–39^ have selective internalization properties in specific cell lines.

## Discussion

Animal venoms are valuable sources of biologically active molecules with selective and specific biological actions. Therefore, they are important tools for the discovery of new drugs and biotechnological products^21^. Herein we present the first description of a scorpion toxin that acts on the intracellular InsP3 receptors. CPP-Ts integrates the list of venomous animal’s toxins that bind specifically to biological targets, which corroborates the potential use of *Ts* venom in biotechnological applications and as a tool to study ion channels and receptors^22,23^.

Scorpion toxins active on Ca^2+^ channels are members of the scorpionic calcine family, which consists of basic peptides with 33 amino acid residues, stabilized by three disulfide bonds, and containing an “inhibitor cystine-knot” (ICK). Ca^2+^ stores in the cytoplasm and nucleus are released through activation of InsP3R and RyR^24–26^. These toxins activate the RyRs, which provide most of the intracellular Ca^2+^ for muscular contraction^19,27–29^. Calcines bind to the RyR1 receptor through their basic amino acid residue agglomerate followed by Ser or Thr [54- **KK**C**KRR**GT-61]^30,31^ (Fig. 1C).

CPP-Ts, on the other hand, has features that are distinct from toxins that act on RyRs. Indeed, it lacks the typical RyR binding basic amino acid region and has instead negatively-charged, neutral, and apolar amino acids [54-**EQCISTGS**–61] (Fig. 1C). In fact, CPP-Ts is most similar to the calcium channel toxin-like BmCa1 from *Mesobuthus martensii* (63% of similarity) and, together, they may form a new subfamily of toxins able to affect Ca^2+^ channel^32,33^ (Fig. 1B).

Studies on Ca^2+^ transient in cardiomyocytes revealed that scorpion calcines induce a significant increase in the Ca^2+^ signal duration and amplitude^18,19^. Likewise, we observed that synthetic CPP-Ts induce Ca^2+^ transient alterations in neonatal rat cardiomyocytes (Fig. 2), thus significantly increasing the frequency of cellular contractions. In addition, CPP-Ts is addressed to the nuclear region (Fig. 3), thereby activating Ca^2+^ signaling through InsP3R (Fig. 4), a previously unrecognized mechanism of scorpionic toxins.

As intracellular Ca^2+^ concentration triggers heart muscle contraction^34,35^, Ca^2+^ channel toxins seem to be active in the cardiac system. Cardiac alterations caused by *Ts* envenoming are one of the most notable and potentially hazardous symptoms^3^. The effect of *Ts* envenoming in the cardiovascular system includes cardiac arrhythmias, arterial hypertension or hypotension, and circulatory failure^36,37^.

Silveira *et al*. showed that the complex effects in the cardiac frequency and contraction force evoked by *Ts* venom were due to simultaneous release of acetylcholine and catecholamine in the postganglionic nerve fibers of the guinea pig heart^37^. However, Teixeira *et al*. concluded that the increased contractility was neurotransmitter independent and caused by a direct effect of *Ts* venom in rat cardiomyocytes^38^. Here we show that *Ts* venom and CPP-Ts have a direct effect on the increase of contraction frequency of neonatal rat cardiomyocytes, corroborating the hypothesis by Teixeira *et al*.^38^. The biodistribution assays suggest that ^99m^Tc-CPP-Ts has a high affinity to heart, liver, and lungs (Fig. 5), which are targets that match with the clinical manifestations frequently observed in Ts scorpion envenomation^3^.

The study of new components from *Ts* venom is important for the understanding of systemic envenoming and may help with the development of alternative anti-scorpion sera^39^. Our research group is involved on the characterization of the immunoreactivity of isolated toxins to foster alternative serum production against venoms^40–46^.

In the current work, the antisera produced against synthetic CPP-Ts neutralized 1 LD_50_ of *Ts* venom in mice (Supplementary Table 1). The inhibition achieved by the anti-CPP-Ts sera shows the relevance of CPP-Ts effects in *Ts* envenoming. Cardiac effects are one of the main causes of death on *Ts* envenoming victims^3^. Since CPP-Ts has a direct cardiac effect, it also has a high potential to be used for alternative sera production, especially if employed along with other components of *Ts* venom.

Toxins belonging to the calcine family show cell internalization properties. Toxins such as IpTxA and Mca fold into compact, mostly hydrophobic molecules, with a cluster of positively-charged basic residues polarized on one side of the molecule that possibly interacts with cell membrane phospholipids^13,18,47^. Since clathrin-mediated endocytosis inhibitors do not affect their internalization process, they probably cross the plasma membrane passively^48^.

We found that CPP-Ts also has cell penetration properties. Within 20 min, CPP-Ts crossed both the cellular and nuclear membranes and concentrated in the nucleus (Fig. 3). Mca concentrated in the cytoplasm after 2 h of incubation and, only between 4 and 24 h, it was localized in the perinuclear or nuclear regions^13^. Therefore, although CPP-Ts and Mca have similar internalization properties, CPP-Ts has a higher nuclear specificity and lower internalization time than Mca. Although the mechanisms of membrane translocation and nuclear internalization of CPP-Ts have not been studied, we believe that it occurs by active means, since CPP-Ts has internalization specificity (Fig. 7) and is negatively charged, which greatly hinders its interaction with the membrane lipids. In contrast, although IpTxA quickly penetrates in the cell cytoplasm, this toxin is not detected in the nucleus^18^.

There is a growing interest regarding CPPs as potential tools for the delivery of “cargo” to their action sites. Indeed, there is an ongoing search for CPPs sub peptides carrying cell internalization properties while lacking pharmacological activity. Mca was the first example of an animal venom peptide with efficient cell penetration properties and has been shown to carry the anticancer drug doxorubicin^49^. Mca analogs lack the pharmacological activity while keeping cell penetration properties^50–53^. Herein we present the CPP-Ts synthetic sub peptide^14–39^ that lacks pharmacological function (Fig. 6), but maintains the nuclear localization property of the original CPP-Ts.

Peptides with cell line-specific internalization properties, which is a characteristic relevant for therapeutic applications, are very rare. The main limitation of classic CPPs such as TAT, Penetratin, Polyarginine, or natural CPPs like Mca, is internalization promiscuity, although the efficiency of non-specific CPPs has been improved^47,54,55^. Surprisingly, the sub peptide^14–39^ has cellular internalization specificity since it translocates through the membranes of primary cultures of its natural targets, such as cardiomyocytes and hepatocytes. Also, the sub peptide^14–39^ shows specificity to cancer cell lines of different human tissues such as melanoma, colon/breast adenocarcinoma, hepatocellular/lung carcinoma, and prostate carcinoma. Remarkably, CPP-Ts was not internalized by the various normal cell lines tested herein (Fig. 7). The mechanism of specificity will need further investigation.

Crotamine-derived nucleolar-targeting peptides translocate rapidly and efficiently to the nucleus of cells that actively proliferate at a given G1/S cell cycle phase and bind to centrosomes and chromosomes^56^. It is possible that a similar mechanism is taking place for the CPP-Ts synthetic sub peptide^14–39^ although, given its negative net charge (as opposed to the positively charged crotamine analogs and Mca), it is also possible to envisage an entirely different nuclear entrance system such as a specific receptor. Future research is needed to clarify this matter.

The nuclear internalization of CPP-Ts observed in all cancer cell lines tested positions the peptide as a very promising tool for the delivery of antitumor drugs, given the nuclear sensitivity for drug-induced DNA damage^57^ and the lack of specificity of the currently available drugs. As the most widely used anticancer drugs belong to the cationic group of anthracyclines^58^, the negative charge of CPP-Ts provides a great advantage because the noncovalent binding of positively charged molecules is considered safer and more effective for drug delivery in blood circulation^59^. Such a specific nuclear drug delivery tool may increase therapeutic efficacy and minimize side effects in cancer therapy. Further studies on the cellular internalization properties of CPP-Ts and efficiency in the transport of molecules should reveal the potential of this toxin as a new CPP for drug delivery in the cellular nucleus of cancer cells.

Concluding, the CPP-Ts that we described here is the first characterized scorpion toxin active in nuclear InsP3R. Its action involves intracellular Ca^2+^ release and consequent alteration in the cardiac frequency, thus explaining the symptomatology of *Ts* envenomation. This natural peptide presents selective internalization properties, with specific nuclear addressing. In comparison with other known natural CPPs^19,47^, CPP-Ts is the quickest to reach the nucleus, showing high nuclear specificity. The ability of CPP-Ts to be internalized by cancer cells and not by normal cell lines as well as its nuclear addressing property make this peptide a potential intranuclear delivery tool to target cancerous cells.

## Methods

### Experimental animals

Neonatal Wistar rats (1–3 days old, 5–7 g) and female Swiss CF1 mice (4–5 weeks old, 18–22 g or 6–8 weeks old, 24–28 g), from the animal care facilities (CEBIO) of the Federal University of Minas Gerais (UFMG), were used. Adult female New Zealand white rabbits (12 weeks old, 2.5 Kg) were obtained from the Animal Facilities Center of the School of Veterinary Medicine, UFMG. All animals had free access to water and food and were kept under controlled environmental conditions. Animal experiments were performed according to the Brazilian Council for Animal Care guidelines and approved by the Ethics Committee of UFMG (Comissão de Ética no Uso de Animais - CEUA, Protocol number 145/2014, and 05/2016).

### Scorpions, RNA and venom extraction

*Ts* scorpions were collected as previously described^40^. Scorpion venom was extracted from 40 female scorpions by electrical stimulation^40^. Protein concentration was measured^60^ and the venom samples were stored at −20 °C.

RNA extraction was performed two days later. The telson containing venom glands was removed and triturated in TRI reagent (Sigma-Aldrich, MO, USA) to isolate RNA following a previously described protocol^61^. RNA quality was evaluated by electrophoresis in a 2.0% agarose gel, quantified in Qubit 2.0 Fluorometer (Life Technologies, MD, USA), and stored at −80°C until cDNA library construction.

### Library construction and RNA sequencing

The RNA library was assembled from total *Ts* telson RNA, using the TruSeq RNA Sample Preparation Kit v2 (Illumina, CA, USA), according to the manufacturer’s instructions. The library was sequenced in a paired-end strategy, using the Reagent kit v3 600 cycles (2 × 300) and run in the Illumina Miseq sequencer (Illumina, CA, USA).

The obtained paired-end reads were trimmed using Prinseq-Lite 0.20.4^62^ using 30 as a quality score threshold. Reads shorter than 40 bp were also excluded. The trimmed sequences were assembled *de novo* using the Trinity assembler^63^.

### Sequence computational analysis

A Blast search was performed against the obtained sequences using a Ca^2+^ toxin database with the BLASTx (Basic Local Alignment Search Tool) standalone package (http://www.ncbi.nlm.nih.gov/books/NBK52640/). The database was obtained from NCBI using the following terms: [*calciumtoxin OR calcium toxin NOT actin NOT transmembrane NOT chloro*[*Title*] *NOT potassium*[*Title*] *NOT sodium*[*Title*] *NOT Escherichia coli*[*Title*]].

The obtained sequence had its identity confirmed by a BLASTx search against the UniProtKB/Swiss-Prot database. Amino acid sequence was analyzed as previously described^40^, and disulfide bonds were predicted by DISULFIND server (http://disulfind.dsi.unifi.it/). BLASTp search^64^ was used to find the sequences that most closely matched CPP-Ts, which were then aligned using the ClusalW on BioEdit Software^65^. A second alignment, using the same methods, was performed using other well-characterized toxins belonging to the scorpionic calcines family.

To verify the subcellular location of CPP-Ts, we performed an *in silico* prediction using the software PSORT II (http://psort.hgc.jp/form2.html). Mature CPP-Ts sequence, in addition to six sub peptides (21–26 residues in length), were analyzed.

### Peptide synthesis

The CPP-Ts peptide containing 45 amino acid residues and three properly positioned disulfide bonds and the CPP-Ts sub peptide containing 26 residues^14–39^ were chemically synthesized by LifeTein, LLC (New Jersey, USA).

### Primary culture, cell lines and cell culture

Neonatal cardiomyocytes were freshly isolated from Wistar rats (n = 12, 1–3 days old, 5–7 g) as previously described^66^, and used for transient Ca^2+^ analysis and internalization assays. Rat hepatocytes primary culture was obtained as previously described^67^. Cell lines HFF-1 (BCRJ 0275), MRC-5 (BCRJ 0180), MDA-MB-231 (BCRJ 0164), A549 (BCRJ 0033), HEP G2 (0103), and HEK-293 (BCRJ 0009) were purchased from Rio de Janeiro Cell Bank (BCRJ, Federal University of Rio de Janeiro, Brazil). Cell lines HUV-EC-C (ATCC CRL-1730), Caco-2 (ATCC HTB-37), DU 145 (ATCC HTB-81), and SK-MEL-188 (CVCL_6098 - Memorial Sloan-Kettering Cancer Center) were obtained from Dr. Adriana Abalen (Federal University of Minas Gerais). Cell line BHK-21 (BCRJ 0050) was obtained from Dr. Rodrigo Rezende (Federal University of Minas Gerais), and MDCK (BCRJ 0171) was obtained from Dr. Francisco C. F. Lobato (Federal University of Minas Gerais). All cell lines were cultured in DMEM medium (Sigma-Aldrich D6429, MO, USA) supplemented with 10% fetal bovine serum - FBS (LGC, São Paulo, Brazil) and 1% Penicillin/Streptomycin (LGC, São Paulo, Brazil), with the exception of HUV-EC-C cells, which were cultured in M199 media (Sigma-Aldrich M5017, MO, USA) supplemented with 20% FBS and 1% Penicillin/Streptomycin. All cell lines were maintained in 5% CO_2_ at 37 °C. All human cell lines used in this study have cell line authentication using the short tandem repeat (STR) profiling technique and were confirmed to be mycoplasma-free.

### Intracellular Ca^2+^ transient analysis

Ca^2+^ transients were monitored in neonatal cardiomyocytes as previously described^68^, using Fluo-4/AM fluorescence (excitation at 488 nm and emission at 515 nm) in the line scan detection mode with intervals of 9 sec immediately after treatment (n = 20 cells per treatment). Treatments were synthetic CPP-Ts (2 µg/ml), sub peptide^14–39^ (2 µg/ml), and *Ts* venom (12.8 µg/ml).

### Immunofluorescence

Neonatal cardiomyocytes were exposed to synthetic CPP-Ts or to the CPP-Ts sub peptide^14–39^ marked with Alexa Fluor 555 Microscale Protein Labeling Kit (555/565 nm; Invitrogen A30007, MA, USA). Confocal immunofluorescence was performed as previously described^68^, using mouse anti-α-actinin antibody 1:150 (Sigma-Aldrich A7811, MO, USA), goat anti-mouse Alexa Fluor 488 1:500 (Invitrogen R37120, MA, USA), and TO-PRO-3 probe 1:800 (Invitrogen T3605, MA, USA) for nuclear staining. For the sub peptide^14–39^ internalization assay in various cell lines, only TO-PRO-3 probe was used for nuclear staining.

Images were collected in a Zeiss Axiovert (Zeiss, CA, USA) confocal microscope. Three lasers were utilized, as follows: excitation at 488 nm and emission at 505–550 nm for Alexa 488; excitation at 568 nm and emission at 585–615 nm for Alexa 555; and excitation at 633 nm and emission at 650 nm for TO-PRO-3.

### InsP3 sponge NLS virus transfection

Neonatal cardiomyocytes were incubated with 100 MOI of InsP3 sponge NLS virus in 1 ml DMEM medium enriched with 10% FBS, which prevents InsP3 binding to the nuclear InsP3R^20^, as previously described^24^. The transfection was verified by mRFP fluorescence emission (excitation at 584 nm and emission at 630 nm) in a fluorescence microscope (Zeiss, CA, USA).

### Biodistribution of synthetic ^99m^Tc-CPP-Ts

#### Radiolabeling procedure, radiochemical purity and *in vitro* stability

Technetium-99m was obtained and radiolabeling and measurements of radiochemical purity and *in vitro* stability of labeled synthetic CPP-Ts were performed as previously described^69^. For biodistribution studies, aliquots of 3.7 MBq of ^99m^Tc-CPP-Ts were injected intravenously into healthy Swiss mice (n = 7, 6–8 weeks old, 24–28 g). After 10, 30, and 60 min post-injection, mice were anesthetized with a mixture of xylazine (15 mg/kg) and ketamine (80 mg/kg). Liver, spleen, kidneys, stomach, heart, lungs, blood, muscle, thyroid, intestine, brain, and pancreas were removed for measuring radioactivity using an automatic scintillation counter^69^. The results were expressed as the percentage of injected dose/g of tissue (%ID/g).

Scintigraphic images were acquired at 10, 30, and 60 min post-injection of 11 MBq ^99m^Tc-CPP-Ts in healthy Swiss mice (n = 3, 6–8 weeks old, 24–28 g). Image acquisition was performed as previously described^69^.

### CPP-Ts biological characterization: anti-CPP-Ts serum production, serum neutralization assay and *in vivo* toxicity

Anti-CPP-Ts serum was produced as previously described^40^. Female New Zealand rabbits (n = 2, 12 weeks old, 2.5 Kg) were injected subcutaneously using two boosters of 100 µg of synthetic CPP-Ts and two boosters of 150 µg of synthetic CPP-Ts at 15-day intervals. Serum titration was performed by ELISA, as previously described^41^. ELISA plates (BD, NJ, USA) were pre-coated with *Ts* venom (5 µg/ml) or synthetic CPP-Ts (5 µg/ml), and anti-CPPTs rabbit serum was titrated using dilutions ranging from 1:100 to 1:102,400. Pre-immune serum was used as control. Absorbance was measured at 492 nm.

*Ts* venom LD_50_ was previously established as 13.2 µg per 20 g mouse^40^. For *in vivo* neutralization assays, *Ts* venom samples (1.5 LD_50_ = 19.80 μg or 2 LD_50_ = 26.72 μg) were incubated for one hour at 37 °C with 150 μL of anti-CPP-Ts rabbit serum or pre-immune serum. After incubation, the samples were applied by subcutaneous injection in randomized groups of Swiss mice (n = 8 per group, 4–5 weeks old, 18–22 g). Within 24 h, the surviving mice were counted. All analyses were single-blinded.

To evaluate the toxicity of CPP-Ts in live animals, a single high dose of synthetic CPP-Ts (72.5 µg, equivalent to 5.5 LD_50_ of *Ts* venom) was subcutaneously injected in Swiss mice (n = 8, 4–5 weeks old, 18–22 g). Animals were observed and the surviving ones were counted within 24 h.

### CPP-Ts internalization assay in various cell lines

We used the following cells for this assay: Rat neonatal cardiomyocytes primary culture; Rat hepatocytes primary culture; HFF-1 – human skin fibroblast; MRC-5 – human lung fibroblast; HUV-EC-C – human vascular endothelium; HEK-293 – human embryonic kidney; BHK-21 – hamster kidney; MDCK – epithelial canine kidney; HEP-G2 – human hepatocellular carcinoma; MDA-MB-231 – human breast adenocarcinoma; A549 – human lung carcinoma; Caco-2 – human colorectal adenocarcinoma; DU 145 – human prostate carcinoma; and SK-MEL-188 – human melanoma.

Cells were plated in coverslips at a confluence of 70–80% and submitted to the internalization assay. For the assay, cells were treated with sub peptide^14–39^ (20 µg/ml) for 1 h. After that, cells were washed and fixed with paraformaldehyde 4% (v/v). The immunofluorescence protocol was followed as described (see section: Immunofluorescence). Images were collected in a confocal microscope (Zeiss, CA, USA) (n = 300 cells).

### Statistical analyses

Data were expressed as mean ± S.E.M. Normality and equal variance were evaluated by Shapiro-Wilk and Levene’s tests, respectively. Means were compared by repeated-measures analysis of variance followed by Mauchly sphericity test or One-way ANOVA. In case of multiple comparisons, a post hoc Bonferroni correction was used. Significance level was set at 0.05 and tests were performed two-sided when possible. All data were analyzed by GraphPad PRISM version 5.00 software (La Jolla, CA, USA) and exact p values were calculated using R (version 3.3.0). Sample size calculations were performed using G Power version 3.1. (Supplementary Data 1).

## Electronic supplementary material

Supplementary Dataset 1

## Data Availability

All the data supporting the findings of this study are included in the article and its Supplementary Information files or are available from the corresponding author upon request. CPP-Ts cDNA and protein sequences are available in GenBank database under the accession number MH061344.
