# Supplementary material for: CPP-Ts: a new intracellular calcium channel modulator and a promising tool for drug delivery in cancer cells
Source: Sci Rep. 2018 Oct 3;8:14739. doi: 10.1038/s41598-018-33133-3 (PMC6170434; doi:10.1038/s41598-018-33133-3)
Supplement: Supplementary file 1 — Supplementary Dataset 1 [file 41598_2018_33133_MOESM1_ESM.docx]

**Supplementary Material**

**CPP-Ts: an intracellular calcium channel modulator and a promising tool for delivery in cancer cells**

Bárbara Bruna Ribeiro de Oliveira-Mendes^a^; Carolina Campolina Rebello Horta^b,^; Anderson Oliveira do Carmo^a^; Gabriela Lago Biscoto^a^; Douglas Ferreira Sales-Medina^a^; Hortênsia Gomes Leal^a^; Pedro Ferreira Pinto Brandão-Dias^a^; Sued Eustáquio Mendes Miranda^c^; Carla Jeane Aguiar^d^; Valbert Nascimento Cardoso^c^; André Luis Branco de Barros^c^; Carlos Chávez-Olortégui^e^; M. Fátima Leite^d^; Evanguedes Kalapothakis^a*^


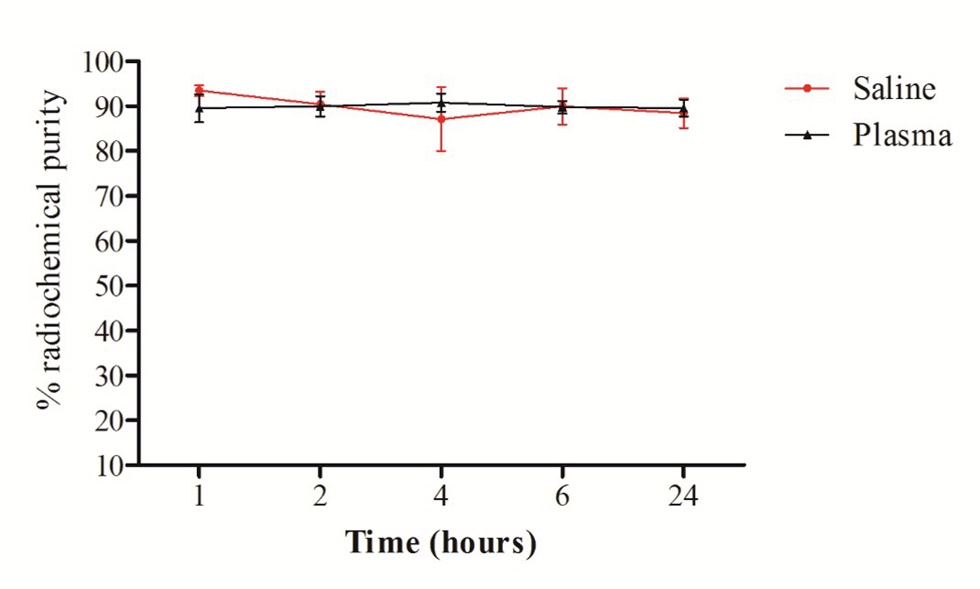


**Supplementary Figure 1. *In vitro* stability of ^99m^Tc-CPP-Ts**. Stability of the complex CPP-Ts with thecnetium-99m as a function of time in the presence of saline, at room temperature, and in the presence of plasma, at 37°C.


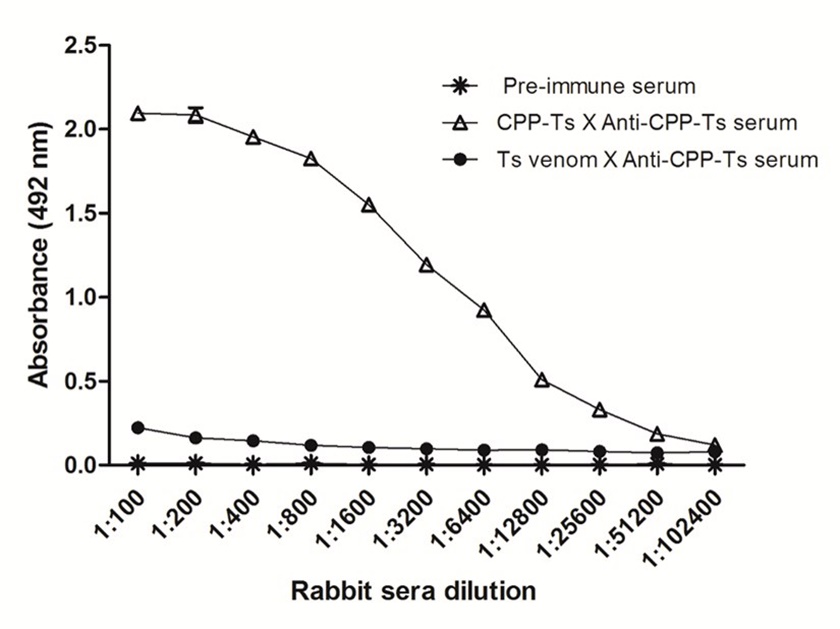


**Supplementary Figure 2. *In vitro* rabbit anti-CPP-Ts serum reactivity.** Reactivity of rabbit anti-synthetic CPP-Ts serum with synthetic CPP-Ts (Δ) or *Ts* venom (•). Pre-immune rabbit serum (*) was used as negative control. ELISA plates were coated with CPP-Ts (5 μg/ml) or *Ts* venom (5 μg/ml). Rabbit anti-synthetic CPP-Ts serum was diluted from 1:100 to 1:102,400. A492 values shown are the means ± S.E.M. of duplicates from three independent experiments.

**
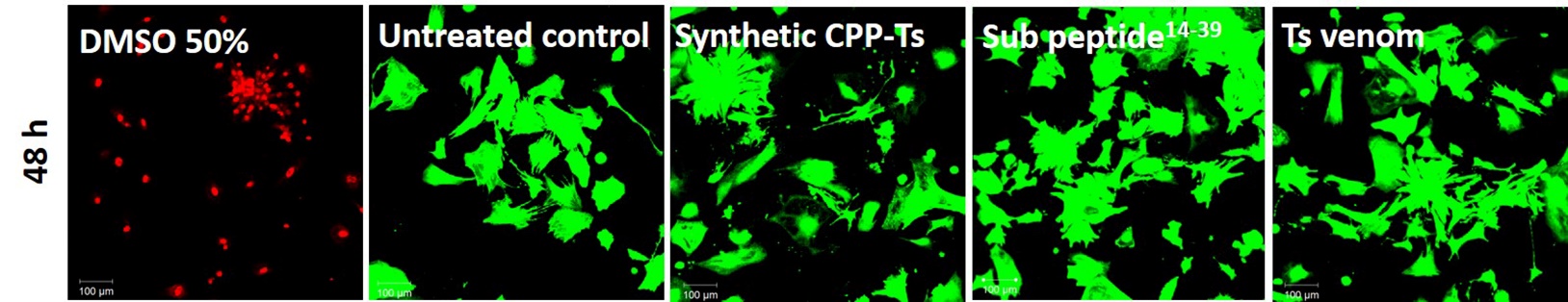
**

**Supplementary Figure 3. Cytotoxicity assay using synthetic CPP-Ts, sub peptide^14-39^ and *Ts* venom in cardiomyocytes.** Neonatal rat cardiomyocytes were treated for 48 h with *Ts* venom (12.8 µg), synthetic CPP-Ts (2 µg) or sub peptide^14-39^ (2 µg) and submitted to LIVE/DEAD Viability/Cytotoxicity kit for mammalian cells (Molecular Probes L3224, OR, USA), used according to the manufacturer’s protocol. Representative images in confocal microscopy show nuclei from dead cells marked in red, and live cells marked in green. Cells treated with DMSO 50% (v/v) were used as cell death control. All the treatments used presented no cytotoxicity to cardiomyocytes (n = 300 cells per treatment).

**Supplementary Table 1. In vivo serum neutralization assays in mice**

| **Serum tested** | ***Ts* venom**  **dose** | **Animals per group** | **Death after 24h** | **Serum neutralization capacity** |
| --- | --- | --- | --- | --- |
| Pre-immune | 2 LD_50_ | 8 | 8 | 0% |
| Anti-CPP-Ts | 2 LD_50_ | 8 | 4 | 50% |
| Pre-immune | 1.5 LD_50_ | 8 | 8 | 0% |
| Anti-CPP-Ts | 1.5 LD_50_ | 8 | 2 | 75% |

## Supplementary Data 1. Methods Statistical analyses - Sample Size

In order to compare multiple means, sample size was calculated considering the variables alpha (α), power effect, effect size (f) and population size (n). Parameters were set at f=0.45, α=0.05, power=0.8, groups=3 to estimate the number of cardiomyocytes per group (Figures 3, 5 and 7) while f=0.7, α=0.05, power=0.8, groups=4 were used to estimate number of mice used in the ^99m^Tc-CPP-Ts biodistribution assays (Figure 6). Sample size for the serum neutralization assay using mice (Supplementary Table 1) was determined expecting to detect a 0.6 proportion difference between groups with α=0.05, power=0.8, and groups=2.
